# Protocol for chromatin immunoprecipitation of chromatin-binding proteins in *Schizosaccharomyces pombe* using a dual-crosslinking approach

**DOI:** 10.1016/j.xpro.2025.103695

**Published:** 2025-03-13

**Authors:** Jasbeer S. Khanduja, Mo Motamedi

**Affiliations:** 1 Massachusetts General Hospital Krantz Family Center for Cancer Research and Department of Medicine, Harvard Medical School, Charlestown, MA 02129, USA

**Keywords:** ChIP-seq, Molecular Biology, Chromatin immunoprecipitation, ChIP

## Abstract

Single-crosslink chromatin immunoprecipitation (ChIP) is often ineffective at mapping the binding sites of chromatin-binding proteins that indirectly interact with DNA. Here, we present a protocol to map the genomic occupancy of different chromatin regulators and an RNA exosome adapter subunit in *Schizosaccharomyces pombe* using dual-crosslinking ChIP. We describe steps for cell growth, dual-crosslinking, cell lysis, sonification, and immunoprecipitation. We then detail procedures for washing, crosslink reversal, and DNA purification for downstream analysis using ChIP-qPCR and ChIP sequencing.

For complete details on the use and execution of this protocol, please refer to Khanduja et al.^1^

## Highlights

• Steps for lysing yeast cells by mechanical force using glass beads
• Instructions for double crosslinking and chromatin preparation
• Guidance on immunoprecipitation, washes, and DNA purification
• Procedures for troubleshooting and improving efficiency

## Abstract (editor highlights)

Publisher’s note: Undertaking any experimental protocol requires adherence to local institutional guidelines for laboratory safety and ethics.

## Graphical abstract

**Figure:**
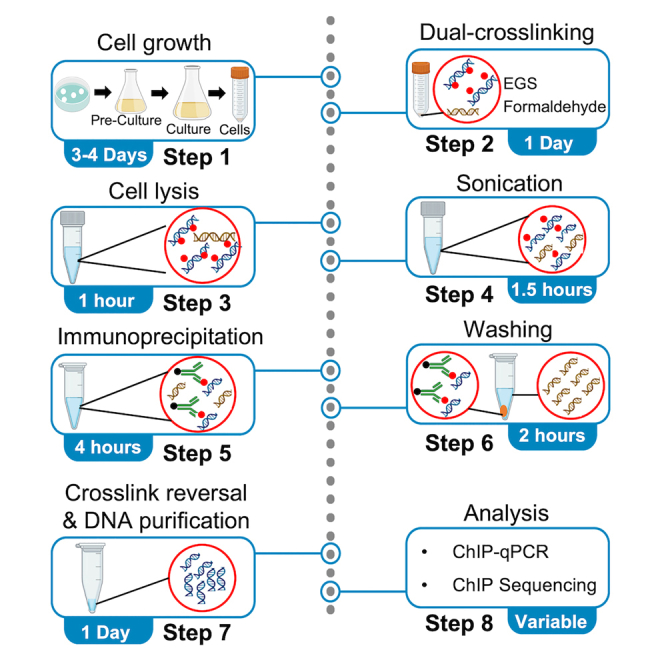

## Before you begin

Chromatin immunoprecipitation (ChIP) is a well-established technique that has enabled the mapping of histone tail modifications and the binding sites of chromatin-associated proteins at specific genomic loci or throughout the genome using ChIP followed by quantitative PCR (ChIP-qPCR) or ChIP followed by deep sequencing (ChIP-seq), respectively. These techniques provide quantitative information about the occurrence of a given histone mark or the localization of a chromatin-binding protein to a given DNA sequence (ChIP-qPCR) or DNA sequences throughout the genome (ChIP-seq). The ChIP assay involves four steps: (1) crosslinking of chromatin-binding proteins to DNA, (2) chromatin fragmentation, (3) immunoprecipitation of target protein using a high-specificity antibody, and (4) identification and quantification of immunoprecipitated DNA using qPCR or next-generation sequencing. Apart from the antibody specificity, the success of a ChIP assay depends on the efficient crosslinking of proteins of interest to DNA. A single crosslink approach using formaldehyde, which crosslinks proteins with DNA within its short spacer arm length of 2Å, has been useful in mapping the binding sites of proteins that directly bind to DNA such as transcription factors, histones, and its post-translational modifications. However, formaldehyde inefficiently crosslinks chromatin-binding proteins which do not interact with DNA directly, such as transcriptional coactivators and corepressors, chromatin regulators, and proteins that transiently interact with chromatin. These complexes often interact with DNA indirectly and are recruited to chromatin as a part of multi-subunit complexes by binding to a histone modification, and/or interacting with a DNA- or RNA-binding protein. The ChIP profiling of the subunits of these complexes (which indirectly interact with DNA) is facilitated by stabilizing protein-protein interactions using different protein crosslinkers with longer arms.^2^^,^^3^^,^^4^^,^^5^

A dual-crosslinking approach was developed to facilitate efficient ChIP of proteins indirectly interacting with DNA.^2^^,^^3^^,^^4^^,^^5^ The dual-crosslinking involves stabilization of protein-protein interactions using an amine-reactive, homo-bifunctional crosslinker with variable spacer arm lengths (7.7–16.1 Å) first, followed by crosslinking of proteins to DNA using formaldehyde.^2^^,^^3^^,^^4^ This approach has been used successfully for the ChIP of transcription factors,^4^^,^^6^^,^^7^ RNA polymerase and its regulators^7^^,^^8^^,^^9^ and corepressors,^10^ and chromatin modifiers.^11^^,^^12^^,^^13^^,^^14^

Previous studies comparing different crosslinkers for a dual-crosslinking ChIP suggested that the optimal protein-protein crosslinker for each target protein should be empirically determined.^3^^,^^4^ EGS has the longest spacer arm length (16.1 Å) of the protein-protein crosslinkers tested for use in the dual-crosslinking protocol, enabling crosslinking of proteins at longer distances from the proteins in immediate contact with the DNA in chromatin.^3^^,^^4^ Our dual-crosslinking protocol is based on a published work demonstrating efficient ChIP of several heterochromatin proteins using EGS in *S. pombe*.^15^ Here we present the dual-crosslinking ChIP protocol for efficient and reproducible ChIP of several proteins including 3x-FLAG-tagged H3K9 methyltransferase, Clr4/SUV39H, the H3K9 deacetylase, Clr3, and a subunit (Red1) of the RNA quality control complex, MTREC (Mtl1 Red1 Core)/NURS (nuclear RNA silencing) from log-phase *S. pombe* cells. Overall, our dual-crosslinking approach using formaldehyde and (ethylene glycol bis (succinimidyl succinate)) EGS provides a highly reproducible and efficient method for the ChIP of proteins refractory to the conventional (formaldehyde-only) ChIP. Moreover, this dual-crosslinking ChIP enabled the fine-mapping of protein binding sites by yielding a high signal-to-noise ratio.

The dual-crosslinking ChIP allows for efficient ChIP of proteins indirectly interacting with DNA which are either refractory to or have a low signal-to-noise ratio in ChIP using formaldehyde crosslinking alone. In the dual-crosslinking ChIP approach, stabilization of protein-protein interactions first by a bifunctional crosslinker followed by crosslinking of proteins in proximity to DNA using formaldehyde leads to reproducible detection of the genomic localization of biological regulators that are not in direct contact with the underlying DNA. In contrast, crosslinking of the factors indirectly interacting with DNA using formaldehyde alone will result in low and variable crosslinking and consequently, a low signal-to-noise ratio in ChIP.

Before starting the ChIP experiment, crosslink the chromatin in yeast cells, establish optimal sonication conditions for chromatin fragmentation and confirm antibody specificity.

### Cell growth and pre-inoculum preparation

**Timing: 3–4 days**

1. Streak yeast strains from stocks stored in the −80°C freezer on yeast extract with adenine (YEA)-agar plates supplemented with appropriate antibiotics and incubate plates at 32°C for 2–3 days until well-isolated single colonies appear.
2. Select a well-isolated colony for each strain and inoculate in 7 mL of liquid YEA media to initiate pre-cultures. Incubate at 32°C on a roller drum for 12–16 h.

### Double crosslinking for ChIP

**Timing: 1 day**

3. Inoculation of cultures for crosslinking.
  a. Measure the OD_600_ of the pre-cultures and inoculate them in 100 mL of YEA media at a starting OD_600_ between 0.01-0.02.
  b. Incubate the cultures overnight at 32°C while shaking at 200 rpm.

***Note:*** Allow for at least 6–8 doublings of the cultures. In our hands, the wild-type yeast cells in the YEA medium have a doubling time of roughly 2.5 h at 32°C. Ideally, user should determine the doubling time of each yeast strain and accordingly inoculate the cell cultures for crosslinking.

4. Harvesting of cells for crosslinking of chromatin.
  a. Next day, when OD_600_ of the cultures has reached 2.2 to 2.5, transfer cells to pre-labeled 50 mL conical tubes.
    **CRITICAL:** If the OD_600_ of the cultures is > 2.5, then it is recommended to re-inoculate the cultures as explained in Step 3 above.
  b. Spin down the cells at 4400 rpm for 2 min at room temperature.
  c. Discard the supernatant and drain the residual-liquid by inverting the tubes on paper towels.
  d. Add 15 mL 1X PBS (at room temperature) to the tubes and mix by gentle vortexing.
  e. Spin down the cells at 4,400 rpm for 2 min at room temperature and discard the supernatant.
    **CRITICAL:** Do not use Tris buffers for washing as they contain primary amines. Washing with PBS is required as even the traces of primary amines from the leftover YEA media will react with the crosslinker, rendering it ineffective for efficient crosslinking of chromatin.
  f. Wash the cells once again with 15mL 1X PBS and drain the residual-liquid by inverting the tubes on paper towels.
5. Crosslinking of chromatin. Troubleshooting - problem 2.
  a. Add 4.6 mL of 1X PBS and resuspend the cells by gentle vortexing. At this step, the total volume of resuspended cells should be 6 mL.
  b. Add 60 μL of 150 mM EGS stock solution (to achieve a final concentration of 1.5 mM of EGS) to the resuspended cells.
    **CRITICAL:** Add EGS stock solutions directly into the resuspended cells and quickly mix by gentle swirling. Do not release EGS stock solution on the walls of 50 mL conical tubes as it may precipitate resulting in inefficient and variability in crosslinking of chromatin.
    ***Note:*** EGS is moisture-sensitive and its crosslinking efficiency might decrease upon exposures to environment over time. We do not recommend aliquoting the EGS stock as it is moisture-sensitive. We recommend the user to follow the manufacturer’s guide.
  c. Incubate the tubes horizontally on an orbital shaker for 30 min with shaking at a low speed.
    ***Note:*** Ensure the cap of the 50 mL tubes is tightly secured. The speed of the orbital shaker should be adjusted such that the cell resuspension gently moves inside the tubes from end to end.
  d. After 30 min, add 162 μL of 37% formaldehyde stock solution to a final concentration of 1% formaldehyde in the resuspended cells.
  e. Incubate the tubes horizontally on an orbital shaker for 30 min with shaking at a low speed.
    ***Note:*** Shorter incubation times (less than 30 min) during crosslinking with formaldehyde are possible; however, we recommend that the user determine the optimal crosslinking time for their protein of interest empirically. We found 30 min incubation for crosslinking with formaldehyde works well for the ChIP of the fission yeast heterochromatin proteins.
    **CRITICAL:** Always use fresh formaldehyde stock. On the day of crosslinking, use formaldehyde from a new ampoule containing 37% formaldehyde stock. Troubleshooting - problems 2 and 3.
    ***Note:*** All steps involving formaldehyde should be done in the fumehood with appropriate personal protective equipment. Before using formaldehyde refer to the MSDS for precautions and best handling practices.
  f. To stop the crosslinking reaction, add 265 μL of 2.5 M glycine stock solution (final concentration of 110 mM glycine) in the resuspended cells. Incubate the tubes horizontally on an orbital shaker for 5 min with shaking at a low speed.
6. Washing of cells and storage of cell pellets.
  a. Spin down the cells at 4,400 rpm for 2 min at 4°C and discard the supernatant in the fume hood in a biohazard container for formaldehyde disposal.
  b. Add 20 mL of ice-cold 1X TBS to each tube, mix by vortexing, and spin down the cells at 4,400 rpm for 2 min at 4°C. Discard the supernatant.
  c. Wash the pellets again with 20 mL of ice-cold 1X TBS as in Steps 6a and 6b.
  d. Resuspend the cell pellets in 1 mL of ice-cold 1X TBS, transfer to a pre-weighed and labeled 1.5 mL screw-cap conical tube using a 1 mL micropipette.
  e. Spin down the cells at 10,000 rpm for 2 min at 4°C, remove the supernatant with a micropipette, weigh the tubes and note the wet pellet weight on the tubes.
  f. Flash-freeze the cell pellets in liquid nitrogen and store them at −80°C for future use.

## Key resources table

**Table:** 

REAGENT or RESOURCE	SOURCE	IDENTIFIER
**Antibodies**		

Monoclonal anti-FLAG M2 antibody produced in mouse (Use 1 μg antibody/120 μL cell lysate)	MilliporeSigma	F1804-1MG
Chemicals		
Trizma base	Sigma-Aldrich	T1503
Ethylenediaminetetraacetic acid disodium salt dihydrate	Sigma-Aldrich	E5134-500G
Sodium dodecyl sulfate	Sigma-Aldrich	L3771-500G
Lithium chloride	Sigma-Aldrich	62476-500G
Triton X-100	Sigma-Aldrich	X100-100ML
Sodium deoxycholate	Sigma-Aldrich	D6750-100G
Sodium chloride	Sigma-Aldrich	746398-1KG
HEPES	Sigma-Aldrich	H3375-500G
PMSF	Thermo Fisher Scientific	36978
Glycine	Sigma-Aldrich	G7126-5KG
Electron Microscopy Sciences formaldehyde 37% microfiltered	Fisher Scientific	50-980-485
Quick Start Bradford 1X Dye Reagent	Bio-Rad	500-0205
Yeast extract	Fisher Scientific	BP1422-2
Adenine	Sigma-Aldrich	A8626-100G
D-(+)-Glucose	Sigma-Aldrich	G8270-1KG
EGS (ethylene glycol bis(succinimidyl succinate))	Thermo Scientific	21565
Electron Microscopy Sciences formaldehyde 37%, microfiltered	Fisher Scientific	50-980-485
Dynabeads Protein G	Thermo Fisher Scientific	10004D
Glycogen	MilliporeSigma	10901393001
Proteinase K solution, RNA grade	Invitrogen	25530-049
cOmplete EDTA-free protease inhibitor	MilliporeSigma	5056489001
Ribonuclease A from bovine pancreas	MilliporeSigma	R4875-500MG
1 kb Plus DNA ladder	Invitrogen	10787-018
Phenol:Chloroform:Isoamyl alcohol 25:24:1, saturated with 10 mM Tris pH 8.0 EDTA 1 mM	MilliporeSigma	P2069-100mL
Chloroform	Fisher Scientific	C298-500

**Experimental models: Organisms/strains**		

*S. pombe* strains	Khanduja et al.^1^	

**Oligonucleotides**		

Primers	Khanduja et al.^1^	

**Other**		

1.5 mL microcentrifuge tubes without caps, polystyrene	Caplugs Evergreen	214-3721-010
Stopper caps for 11 mm tubes, natural	Caplugs Evergreen	300-2911-020
1.5 mL conical screw cap tube, natural	USA Scientific	1415-8700
Falcon 5 mL round bottom polypropylene tubes	Corning	352063
Falcon 50 mL polypropylene conical tubes	Corning	352070
0.5 mm diameter glass beads	BioSpec Products	11079105
BrandTech Brand 0.5 mL thin wall PCR tubes	Fisher Scientific	1388261
23 G x 1 in. BD PrecisionGlide needle	BD	305145
DNA LoBind tubes, 1.5 mL	Eppendorf	30108418
Fisherbrand disposable cuvettes	Fisher Scientific	14-955-127
Eppendorf centrifuge 5810R	Eppendorf	5811000015
MagNA Lyser Instrument	Roche	03-358-968-001
Q Sonica 800R1	Qsonica	Q800R1-110
Q Sonica Chiller	Qsonica	#4905
Sample tube holder (8 slots)	Qsonica	#440
DynaMag-2 magnet	Thermo Fisher Scientific	12321D
Tube rotator	VWR	10136-084
Rotisserie plate assembly for 36 1.5/2.0 mL tubes	VWR	13916-830
Eppendorf Thermomixer 5350	Eppendorf	NA
NanoDrop 2000c spectrophotometer	Thermo Fisher Scientific	ND-2000

## Materials and equipment

**Table:** YEA (Yeast Extract supplemented with adenine) media

Reagent (stock)	Amount
Yeast Extract	5 g
Glucose	20 g
Adenine	0.225 g
ddH_2_O	1 L

Autoclave and store at room temperature. Take out the media from the autoclave immediately after the sterilization cycle to avoid the caramelization of glucose. This media can be used for up to 2 months if not discolored and is without visible contamination.

**Table:** ChIP Buffer

Reagent (stock)	Final concentration	Volume
HEPES.KOH pH 7.4 (1 M)	50 mM	2.5 mL
Sodium Chloride (5 M)	140 mM	1.4 mL
EDTA pH 8.0 (0.5 M)	1 mM	0.1 mL
Triton X-100 (20%)	1%	2.5 mL
Sodium Deoxycholate (10%)	0.1%	0.5 mL
Protease Inhibitor tablet (1 tablet in 1 mL of sterile water)	N/A	1 mL
Phenyl methyl Sulfonyl Fluoride (PMSF) (0.1 M)	1 mM	0.5 mL
ddH_2_O	N/A	41.5 mL
Total	N/A	50 mL

Make ChIP buffer fresh and keep it on ice throughout the experiment.

***Note:*** Add Protease Inhibitor and PMSF only to the volume of ChIP buffer to be used immediately at each step.

**Table:** Elution Buffer 1

Reagent (stock)	Final concentration	Volume
Tris.HCl pH 8.0 (1 M)	50 mM	50 μL
EDTA pH 8.0 (0.5 M)	10 mM	20 μL
Sodium Dodecyl Sulfate (SDS) (10%)	1%	100 μL
ddH_2_O	N/A	830 μL
Total	N/A	1 mL

Make fresh while the washing steps are in progress, and keep at room temperature.

**Table:** Elution Buffer 2

Reagent (stock)	Final concentration	Volume
Tris.HCl pH 8.0 (1 M)	10 mM	10 μL
EDTA pH 8.0 (0.5 M)	1 mM	2 μL
Sodium Dodecyl Sulfate (SDS) (10%)	0.68%	68 μL
ddH_2_O	N/A	920 μL
Total	N/A	1 mL

Make fresh while the washing steps are in progress and keep at room temperature.

**Table:** Input Buffer

Reagent (stock)	Final concentration	Volume
Tris.HCl pH 8.0 (1 M)	10 mM	10 μL
EDTA pH 8.0 (0.5 M)	1 mM	2 μL
Sodium Dodecyl Sulfate (SDS) (10%)	1%	100 μL
ddH_2_O	N/A	888 μL
Total	N/A	1 mL

Make fresh while elution from beads is in progress.

**Table:** 10x PBS

Reagent (stock)	Amount
Sodium Chloride	80 g
Potassium chloride	2 g
Disodium Hydrogen Phosphate	14.4 g
Potassium dihydrogen Phosphate	2.7 g
Water	900 mL

Adjust pH to 7.4 with HCl and bring up the final volume to one liter with water. Autoclave and store at room temperature. This solution can be stored at room temperature and used for one year.

**Table:** Proteinase K-Glycogen mix

Reagent (stock)	Volume
Proteinase K (20 mg/ml)	20 μL
Glycogen (20 mg/ml)	12 μL
1x T.E.	968 μL

**10x TBS** - Dissolve 24 g Tris and 88 g NaCl in 900 mL of water. Adjust pH to 7.6 with HCl and bring up the final volume to one liter with water. Store at room temperature and can be used for six months or longer.

## Step-by-step method details

### Cell lysate preparation for ChIP

**Timing: 45 min**

In this step, yeast cells are lysed by the mechanical force generated by bead-beating of cells resuspended in lysis buffer using glass beads.

1. Take out the double crosslinked cell pellets from −80°C freezer and thaw them at room temperature for 5 min.
2. After thawing, place the cell pellets on ice and add 500 μL of freshly prepared ChIP buffer.

**CRITICAL:** The weight of the frozen crosslinked cell pellet should be between 0.12-0.14 g for efficient cell lysis by bead beating in 1.5 mL conical screw cap tubes using the lysis protocol in steps 4-7. The user should optimize the cell lysis conditions for higher cell pellet weight, alternative tube volume, and different ChIP buffer compositions. Troubleshooting - problem 1.

3. Vortex the tubes to resuspend the cell pellets in the ChIP buffer homogenously.
4. Add 1 “scoop” of cold glass beads to each tube in the cold room.

***Note:*** Here scoop is a 0.5 mL PCR tube (Brand Tech #1388261) full of glass beads.

5. Load the tubes in MagNA Lyser and bead beat the cells at 6000 rpm for 30 s at 4°C.

**CRITICAL:** The bead beating cycles should be kept short and cell lysate should be incubated on ice after each round of bead beating to avoid heat denaturation of cell lysate.

6. Take out the tubes from MagNA Lyser and place them in ice for 3 min.
7. Repeat steps 5 and 6 three more times. Remove the tubes from MagNA Lyser and keep them in ice.

***Note:*** At this stage, fill chilled Milli Q water (or equivalent) in the Q Sonica 800R1 water bath sonicator up to the recommended level and switch on the attached circulating water chiller. Ensure that the set temperature on the chiller is 4°C.

8. Using a needle (BD #305145) punch a hole at the bottom of 1.5 mL tube containing the lysed cells and place it in a pre-chilled 5 mL polypropylene tube on ice. Repeat this for all the tubes, one at a time.
9. Spin the 5 mL tubes using the swing bucket rotor in the Eppendorf 5810R centrifuge at 4000 rpm for 1 min at 4°C.
10. Take out the 5 mL tubes from the centrifuge and place them in ice. Discard the 1.5 mL screw-cap tubes containing the glass beads in a biohazard disposal container. Resuspend the cell lysate in the 5 mL tubes by gently pipetting with a P1000 micropipette.
11. Transfer the cell lysate to 1.5 mL pre-chilled polystyrene tubes (Caplugs Evergreen #214-3721-010) on ice and cap the tubes.

### Chromatin shearing by sonication of the lysates

**Timing: 1 h 30 min (for 8 samples using an 8-slot tube rack)**

In this step, chromatin in yeast cell lysate is sheared by sonication in a cup-horn sonicator with an attached circulating water bath. Using this sonicator results in even chromatin shearing of all samples at 4°C and minimizes heat denaturation of protein epitopes during this process. With optimized sonication settings (see step 13 below), the cup-horn water bath sonicator fragments chromatin in *S. pombe* cell lysates to a size range of 100–500 bp with a peak between 150-300 bp, a desirable size range for downstream qPCR and DNA deep sequencing applications. Troubleshooting - problem 2.

12. Load the 1.5 mL polystyrene sonication tubes in the tube rack and attach the rack to the sonicator rack holder. Ensure that the water level in the sonicator is not above the top-margin of the lower plate of the sample holder rack.
13. Attach the sonicator controller, power it on, and adjust the sonication parameters as:

  Pulse on: 20 s; Pulse off: 40 s; Time: 30 min; Amplitude: 100%

14. Hit the start button and observe the sonication process briefly to ensure that:
  a. The water is circulating and the chiller is holding the temperature at 4°C.
  b. The tube rack holder is secure in place and rotating, and the tubes are not touching the sonicator horn.
  c. The water does not splash over the caps of the tubes during the sonication cycle.
15. At the end of the sonication cycle, remove the tube rack from the sonicator and place the tubes in ice.

***Note:*** Record the sonication energy from the controller display at the end of the sonication run.

16. Spin the tubes at 14000 rpm/10 min/4°C. Carefully transfer the supernatant from each tube into a pre-chilled Eppendorf tube on ice.
17. Spin the Eppendorf tubes at 14000 rpm/10 min/4°C. Carefully transfer the supernatant from each tube into a pre-chilled Eppendorf tube on ice.

***Note:*** We do not recommend preparing and storing the yeast cell lysates at −80°C for ChIP later. Users must empirically determine the change in ChIP efficiency of the protein of interest if lysates were stored at −80°C for performing ChIP later. Frozen ChIP lysates must be slowly thawed on ice and any precipitates must be removed by centrifugation (see step 17 above) before Bradford’s assay and immunoprecipitation steps.

18. Aliquot 20 μL of clarified lysate, add 240 μL of 1x T.E. buffer, reverse the DNA-protein crosslinks and purify the DNA following steps 53 to 64 below. Quantify the purified DNA on a nanodrop spectrophotometer, load 2.5–5.0 μg of purified DNA of each sample on a 1% agarose gel along with a suitable DNA size marker, and perform gel electrophoresis and image documentation using established methods.

**Figure 1:**
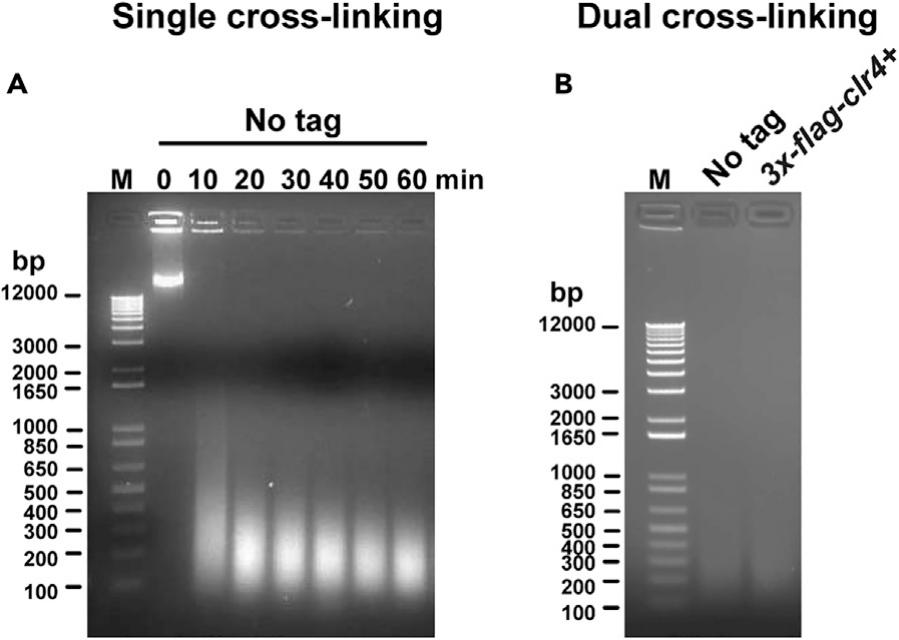
DNA size distribution profile of fragmented chromatin from single and dual-crosslinked cells (A) DNA size distribution profile upon chromatin fragmentation of formaldehyde crosslinked cells at 100% amplitude and the indicated times, with 20 s ON and 40 s OFF cycle, in a cup-horn sonicator with an attached circulating water bath. Cells were zymolase-treated before chromatin shearing in a sonicator. 4 μg of 1Kb Plus DNA ladder in the marker (M) lane, and 5 μg of chromatin (after purification of total cell lysate by phenol: chloroform extraction) were loaded in each lane for agarose gel-electrophoresis. (B) DNA size distribution profile upon chromatin fragmentation of dual-crosslinked cells at 100% amplitude for 30 min, with 20 s ON and 40 s OFF cycle, in a cup-horn sonicator with an attached circulating water bath. Cells were lysed by bead beating before chromatin shearing in a sonicator. 4 μg of 1 kb Plus DNA ladder in the marker (M) lane, and 2.5 μg of chromatin were loaded in each lane for agarose gel-electrophoresis. After purification of clarified cell lysate by phenol: chloroform extraction, 2.5 μg of purified DNA was treated with RNaseA (1 mg/ml) for 1 h at 37°C to degrade any copurifying RNA. After RNaseA treatment the samples were directly used for agarose gel electrophoresis without purification of the DNA again. This resulted in lower band intensity of sheared DNA profile on the agarose gel than expected of 2.5 μg of DNA/lane loaded on the gel.

***Note:*** In our hands chromatin shearing of single and dual-crosslinked cells yielded DNA fragments distributed over 150–500 bp with a peak between 150-300 bp (Figures 1A and 1B).

***Note:*** The cell lysate aliquots for chromatin fragmentation profile can be stored at −80°C, and steps for reversal of crosslinking and purification of DNA can be performed at the end of the experiment along with the ChIP and Input samples. We suggest the user establish the sonication parameters for optimal chromatin fragmentation beforehand and use step 18 to confirm the size distribution of sheared chromatin in each ChIP experiment.

### Quantification of total proteins in the sonicated cell lysates

**Timing: 45 min (for 8 samples)**

In this step, the total amount of proteins in each cell lysate is quantified using the Bradford’s method. Based on this quantitation, a normalized amount of cell lysate is used for each immunoprecipitation reaction.

19. Dilute 2 μL of each sonicated lysate with 18 μL of sterile water in separate PCR tubes.
20. Transfer 2 μL of the diluted sonicated lysates to 1 mL cuvettes and add 18 μL of sterile water.
21. Add 1 mL of Bradford reagent to the cuvettes and mix the solution by pipetting. Let them stand for at least 3 min for the color to develop.
22. Measure protein concentration of sonicated cell lysates using Bradford’s assay in a NanoDrop spectrophotometer.
23. Normalize the protein concentration across the sonicated cell lysates by diluting the concentrated lysates with ChIP buffer. Transfer 500 μL of each normalized lysate into a pre-chilled Eppendorf DNA LoBind tube on ice.
24. Aliquot 10 μL (2%) of each normalized cell lysate into a pre-chilled Eppendorf DNA LoBind tube on ice and label them as Input. Store them at −20°C until the ChIP samples are ready for reversal of crosslinks.

***Note:*** Some protocols, instead of measuring total protein, measure total nucleic acid levels in each sample for normalization. We have found that either strategy works well to normalize samples.

### Washing and pre-equilibration of protein G Dynabeads for the ChIP assay

**Timing: 40 min**

In this step, the Protein G Dynabeads are washed and equilibrated with the ChIP buffer for subsequent use in the pre-clearing and immunoprecipitation steps.

25. Wash Protein G Dynabeads (or other beads suitable for the ChIP assay).
  a. Take 40 μL (10 μL for pre-clearing + 30 μL for immunoprecipitation steps) of Protein G Dynabeads/sample in an Eppendorf tube and incubate it on a magnetic rack for 30 s.
    ***Note:*** For 8 different samples, we will need 320 μL of Protein G Dynabeads. However, we start with Protein G Dynabeads for 9 reactions (360 μL), to account for any loss during washing and pipetting.
  b. Remove the supernatant with a P1000 micropipette and place the tubes with beads on ice.
  c. Add 1 mL of ChIP buffer, cap the Eppendorf tubes, and incubate them on the tube rotator with rotation for 10 min at 4°C.
  d. Take out the tubes from the tube rotator, and place them in the magnetic rack for 30 s.
  e. Remove the supernatant with a P1000 micropipette and wash the Protein G Dynabeads at least two more times with 1 mL of the ChIP buffer.
  f. Resuspend the beads in the original bead volume of ChIP buffer (360 μL) and place the tubes on ice.
    **CRITICAL:** Though sepharose and agarose beads have been used in the past in ChIP assays, we recommend using magnetic beads to minimize the background. User must select the appropriate Protein G or Protein A or Protein G/A beads depending on the antibody used for immunoprecipitation.
    ***Note:*** Users may elect to block the beads (using standard protocols), to minimize the non-specific interactions leading to reduced background noise and increased specificity of target protein-antibody interactions.

### Chromatin immunoprecipitation using protein/epitope tag-specific antibody

**Timing: 6 h (for 8 samples)**

In this step, the genomic DNA bound by the protein of interest is captured by immunoprecipitation using a high-specificity antibody against the protein. If the protein is tagged with an epitope tag (e.g., 3X-FLAG), immunoprecipitation will be performed with a high-specificity antibody against the tag. The target protein-antibody complexes immobilized on inert magnetic beads are selectively retained through multiple washing steps while the non-specifically bound proteins are washed off. An optional pre-clearing step before immunoprecipitation with specific antibodies might help reduce the background associated with beads. In the steps below, we used an anti-FLAG tag antibody to capture 3X-FLAG tagged protein of interest present in the yeast cell lysates. Troubleshooting - problem 3.

#### Pre-clearing

26. Pre-clear each lysate by adding 10 μL of pre-equilibrated Protein G Dynabeads and incubating the Eppendorf tubes on a tube rotator with rotation for 1 h at 4°C in a cold room.

#### Immunoprecipitation

27. Briefly (10 s) spin the Eppendorf tubes in a mini microfuge and place the tubes in a magnetic rack for 30 s to collect the Protein G magnetic beads.
28. Carefully remove the supernatant from each tube into a pre-chilled Eppendorf DNA LoBind tube on ice and add 4 μg of anti-FLAG antibody (1 μg of antibody/120 μl of cell lysate) to each.
29. Incubate the Eppendorf tubes on a tube rotator with rotation for 1 h at 4°C in a cold room.
30. Add 30 μL of pre-equilibrated Protein G Dynabeads to each Eppendorf tube and further incubate them on a tube rotator with rotation for 2 h at 4°C in a cold room.

***Note:*** Longer incubation times (e.g. overnight incubation) with the antibody and beads can be used to optimize the immunoprecipitation efficiency; however, the protein of interest may become susceptible to proteolysis, thus compromising the IP and consequently ChIP efficiency. Immunoprecipitation length could be optimized empirically for each protein.

#### Washing

31. Briefly (10 s) spin the Eppendorf tubes in a mini microfuge and place the tubes in a magnetic rack for 30 s to collect the Protein G magnetic beads.
32. Carefully remove the supernatant without disturbing the beads and discard it. Keep the Eppendorf tubes in ice.
33. Add 1 mL of ice-cold ChIP buffer to each Eppendorf tube and incubate them on a tube rotator with rotation for 5 min at 4°C in a cold room.
34. Spin and remove supernatant (see steps 31 and 32 above).
35. Add 1 mL of ice-cold ChIP buffer to each Eppendorf tube, gently resuspend the beads with a P1000 micropipette, and transfer them to new pre-chilled Eppendorf DNA LoBind tubes on ice. Incubate the Eppendorf tubes on a tube rotator with rotation for 5 min at 4°C in a cold room.

***Note:*** To minimize the background, high salt buffers (concentrations ranging from 140 to 500 mM NaCl) can be used for washing of beads (steps 33-35). However, the effect of high salt buffers on the stability of antigen-antibody complex should be determined beforehand to avoid loss of immunoprecipitated DNA and consequent low and variable ChIP signal in the experiment.

36. Spin and remove supernatant: repeat steps 31-33 once followed by steps 31 and 32 once.
37. Add 1 mL of ice-cold 1x T.E. buffer to each Eppendorf tube and incubate them on a tube rotator with rotation for 5 min at 4°C in a cold room.
38. Spin and remove supernatant (see steps 31 and 32 above).
39. Add 1 mL of ice-cold 1x T.E. buffer to each Eppendorf tube, gently resuspend the beads with a P1000 micropipette, and transfer them to new pre-chilled Eppendorf DNA LoBind tubes on ice.
40. Incubate the Eppendorf tubes on a tube rotator with rotation for 5 min at 4°C in a cold room.
41. Repeat steps 31 and 32 one more time as above, except that at step 32 keep the Eppendorf tubes on a tube rack at room temperature.

#### Elution

42. Add 100 μL of pre-warmed Elution buffer 1 to each tube, resuspend the beads by gentle tapping, and incubate them at 65°C in a thermomixer with shaking at 1000 rpm for 15 min.
43. Briefly spin the Eppendorf tubes in a mini microfuge and place them in a magnetic rack for 30 s to collect the Protein G magnetic beads.
44. Carefully transfer the supernatant to new pre-labeled Eppendorf DNA LoBind tubes at room temperature. This is Elution 1.
45. Add 150 μL of pre-warmed Elution buffer 2 to each Eppendorf tube, resuspend the beads by gentle tapping, and incubate them at 65°C in a thermomixer with shaking at 1000 rpm for 5 min.
46. Repeat step 43.
47. Carefully transfer the supernatant from each Eppendorf tube to the corresponding Eppendorf tubes containing Elution 1 for each sample. The total elution volume for each ChIP sample is 250 μL.
48. Take out the ChIP Inputs from −20°C freezer (from Step 24) and add 240 μL of Input Buffer to each tube. Mix the contents by vortexing briefly.

**Pause Point:** The protocol can be paused at this point and samples can be stored at −80°C/−40°C.

### Reversal of crosslinks and degradation of RNAs and proteins in the ChIP inputs and elutions

**Timing: 15 h 30 min (for 16 samples)**

In this step, protein-DNA crosslinking is reversed by incubating all samples (eluates and inputs) at a high temperature (65°C) and RNA and proteins are degraded by RNaseA and Proteinase K treatment.

49. Incubate the ChIP Elutions and Inputs, from steps 47 and 48 respectively, overnight at 65°C in a thermomixer with shaking at 1000 rpm.
50. Remove the Eppendorf tubes from the thermomixer, briefly (10 s) spin the tubes in a mini microfuge and place them on a tube rack at room temperature.
51. Add 50 μg of RNaseA to each Eppendorf tube, mix by gentle tapping, and incubate them at 37°C for 1 h. After incubation, briefly spin the Eppendorf tubes in a mini microfuge and place them on a tube rack at room temperature.
52. Add 258 μL of Proteinase K-Glycogen mix to each Eppendorf tube, gently vortex, and incubate the samples at 55°C in a thermomixer with shaking at 1000 rpm for 2 h. After incubation, briefly spin the Eppendorf tubes in a mini microfuge and place them on a tube rack at room temperature.

**Pause Point:** The protocol can be paused at this point and samples can be stored at −40°C.

### Purification of ChIP DNA

**Timing: 6 h 30 min (for 16 samples)**

In this step, the ChIP DNA is purified for downstream applications using standard phenol-chloroform extractions and ethanol precipitation.

53. Phenol-Chloroform Extractions.
  a. Add 500 μL of phenol-chloroform-isoamyl alcohol mix (25:24:1) to each Eppendorf tube and mix by inverting the Eppendorf tubes multiple times at room temperature.
  b. Spin the Eppendorf tubes in a centrifuge at 13500 rpm for 12 min at room temperature. Carefully remove the supernatant from each tube into a pre-labeled new Eppendorf DNA LoBind tube.
    ***Note:*** Do not touch the interface while pipetting out the aqueous phase. We generally recover 450–460 μL of aqueous solution at this step.
  c. Add 450 μL of chloroform to each Eppendorf tube and mix by inverting the Eppendorf tubes at room temperature.
  d. Spin the Eppendorf tubes in a centrifuge at 13500 rpm for 12 min at room temperature. Carefully remove the supernatant from each Eppendorf tube into a pre-labeled new Eppendorf DNA LoBind tube.
    ***Note:*** Do not touch the interface while pipetting out the aqueous phase. We generally recover 380–400 μL of aqueous solution at this step.
54. Ethanol Precipitation of ChIP DNA.
  a. Add 50 μL of 4 M LiCl solution and 960 μL of chilled 100% Ethanol to each Eppendorf tube. Mix the solutions by inverting the tubes multiple times and incubate them at −40°C for at least 2 h.
    ***Note:*** Alternatively, samples can be kept at −80°C for ethanol precipitation. Longer incubation periods at −80°C/−40°C for ethanol precipitation are also fine.
    **Pause Point:** The protocol can be paused at this point and samples can be stored at −80°C/−40°C.
  b. Take out the Eppendorf tubes from the −40°C freezer and spin them at 13500 rpm for 15 min at 4°C.
  c. Remove the supernatant with a P1000 micropipette, without disturbing the DNA pellet, and discard it.
  d. Add 500 μL of 70% Ethanol to each Eppendorf tube, gently tap them, and spin them at 13500 rpm for 15 min at 4°C.
  e. Remove the supernatant with a P200 micropipette, without disturbing the DNA pellet, and discard it.
  f. Dry the DNA pellets by keeping the Eppendorf tubes open in a tube rack at 37°C for 30 min.
  g. Resuspend the ChIP DNA pellets in 40 μL of 0.1x T.E. buffer and the Input DNA in 50 μL of 0.1x T.E. buffer. Incubate the Eppendorf tubes at 37°C for 1 h on a thermomixer with shaking at 1000 rpm.
  h. Remove the tubes from the thermomixer, briefly spin them in a mini microfuge, and store the resuspended DNA at −80°C/−40°C for downstream applications.

## Expected outcomes

**Figure 2:**
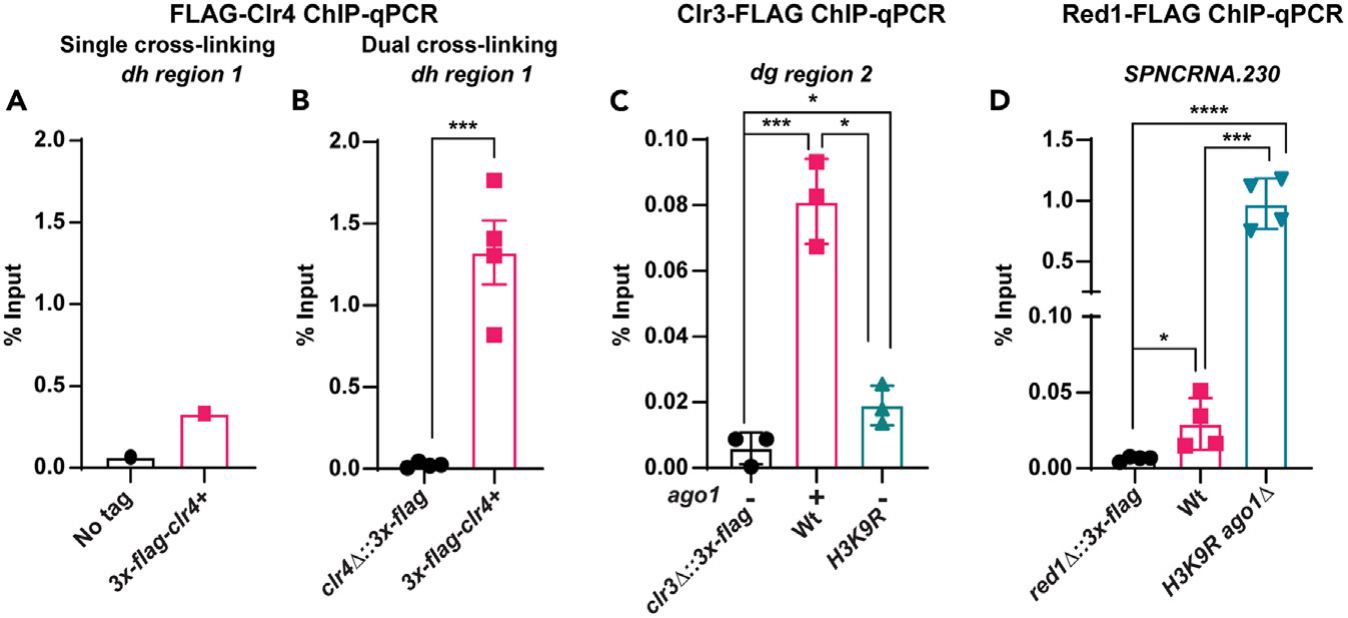
Dual-crosslinking ChIP results in specific enrichment of chromatin binding proteins indirectly interacting with DNA (A) Graph depicting 3XFLAG-Clr4 ChIP-qPCR (mean percent input) at a *dh* sequence in the indicated strains crosslinked using formaldehyde only (single crosslinking). The “no tag” strain served as the background control. A representative graph from two independent experiments is shown. (B) Graph depicting 3XFLAG-Clr4 ChIP-qPCR (mean percent input) at a *dh* sequence in the indicated strains dual-crosslinked using EGS and formaldehyde (double crosslinking). *clr4Δ::3xflag* served as a FLAG background control. Error bars - S.D.; *n* = 4 biological replicates. (C) Graph depicting Clr3-3XFLAG ChIP-qPCR (mean percent input) at a *dg* sequence in the indicated strains dual-crosslinked using EGS and formaldehyde (double crosslinking). *clr3Δ::3xflag* served as a FLAG background control. Error bars - S.D.; *n* = 3 biological replicates. (D) Graph depicting Red1-3XFLAG ChIP-qPCR (mean percent input) at *SPNCRNA.230* in the indicated strains dual-crosslinked using EGS and formaldehyde (double crosslinking). *red1Δ::3xflag* served as a FLAG background control. Error bars - S.D.; *n* = 4 biological replicates. In panels B-D, statistical significance was determined using a two-tailed unpaired Student’s *t*-test. ∗*p* < 0.05; ∗∗∗*p* < 0.001; ∗∗∗∗*p* < 0.0001. In panels B-D, data were reused for figures with permission from.^1^

A successful dual-crosslinking ChIP protocol should result in the enrichment of the proteins indirectly interacting with DNA at the anticipated binding sites in the genome as measured by ChIP-qPCR and ChIP-seq assays. The enrichment of proteins, measured as percent input by ChIP-qPCR, can vary depending on the abundance of a protein of interest, repetitive nature of the DNA sequence bound by the protein (e.g., constitutive heterochromatin proteins), crosslinking efficiency, the nature of the interaction (stable versus weak/transient), and the efficiency of ChIP (governed by the antibody specificity and immunoprecipitation efficacy). Abundant chromatin proteins with stable interaction and efficient ChIP can be expected to show ChIP enrichment of 0.5–2.5% of Input, whereas less abundant and weakly/transiently interacting chromatin proteins are likely to show ChIP enrichment below 0.1% of Input. In our hands, the ChIP of 3XFLAG tagged Clr4 (the sole H3K9 methyltransferase in *S. pombe*) using formaldehyde-only crosslinking resulted in a low and variable ChIP enrichment (Figure 2A and data not shown). Using the optimized dual-crosslinking protocol (employing EGS and Formaldehyde crosslinkers), we achieved specific and reproducible enrichment of 3XFLAG tagged Clr4 at the constitutive heterochromatin (Figure 2B). We also used the dual-crosslinking protocol for reproducible ChIP enrichment of Clr3 (an H3K9 deacetylase) at pericentromeric repeats (Figure 2B). ChIP of Red1 (a structural protein of the MTREC complex), using dual-crosslinking, exhibited noncoding RNA (ncRNA) level-dependent enrichment at *SPNCRNA.230* in the pericentromeric repeats, with higher RNA expression correlating with a higher ChIP signal. Small, but statistically significant, enrichment of Red1 observed in wild-type cells increased significantly in the heterochromatin deficient strain (*H3K9R ago1Δ*; where the steady state level of *SPNCRNA.230* is higher than in the wild-type strain) (Figure 2C). Overall, the dual-crosslinking protocol to ChIP chromatin proteins indirectly interacting with DNA offers a practical/useful approach for mapping the binding sites of proteins refractory to the conventional ChIP using formaldehyde-only crosslinking.

## Quantification and statistical analysis

For qPCR data, statistical significance was determined using a two-tailed unpaired Student’s *t*-test in GraphPad Prism Version 8.3.0 (538). *n* represents the number of biological replicates and error bars show standard deviation (SD). The statistical significance was determined using GraphPad Prism Version 8.3.0, where, ∗*p* < 0.05; ∗∗∗*p* < 0.001; ∗∗∗∗*p* < 0.0001.

## Limitations

The initial step in dual-crosslinking ChIP protocol involves stabilizing protein-protein interactions using a bifunctional crosslinker, with a specific arm length, empirically determined for each protein of interest. Our optimized dual-crosslinking ChIP protocol uses EGS, whose suitability for the ChIP of other proteins using this protocol must be determined. Moreover, EGS is moisture-sensitive; its NHS ester moiety readily hydrolyzes and becomes non-reactive. Using an EGS stock exposed to the environment multiple times might decrease its crosslinking efficiency over time, thus leading to suboptimal performance of the protocol even with the tested proteins. Crosslinking might change the conformation of proteins of interest and affect their reactivity to the antibody being used for immunoprecipitation in the ChIP experiment. The reactivity of crosslinked protein to the ChIP antibody should be tested using western blotting before running the ChIP experiment. Our ChIP protocol uses a cup-horn sonicator, with an attached circulating water bath, for chromatin fragmentation to a uniform size range between 100-500 bp at 4°C. Using alternative instruments, to achieve chromatin shearing can alter the size distribution of fragmented chromatin and the stability of proteins in the lysate impacting the protocol performance.

## Troubleshooting

### Problem 1

Inefficient cell lysis.

The extent of cell lysis governs the amount of chromatin available for fragmentation, the final fragmented chromatin profile, and consequently the success of the ChIP experiment.

### Potential solution

User should determine the cell weight: glass beads: lysis buffer ratio for efficient cell lysis by bead beating the cells in screw-cap conical tubes on their specific equipment. The extent of cell lysis can be determined by visualizing an aliquot of cell lysate using a hemocytometer under a standard microscope and comparing the number of intact cells before and after bead beating.

### Problem 2

Inconsistent chromatin shearing.

A critical step in a successful ChIP assay is shearing the chromatin to a size suitable for efficient IP and evaluation by downstream assays. Sub-optimal or excessive crosslinking of chromatin can alter the size distribution of sheared chromatin, compromising the success of the ChIP assay. Physical parameters such as sonication amplitude, cycle-on and off times, and circulating water temperature also can influence chromatin shearing and protein stability. Moreover, using a sonicator with a worn-out horn for chromatin shearing will result in an altered chromatin fragmentation profile and may lead to the failure of even a validated ChIP experiment.

### Potential solution

• For optimal crosslinking see the “Critical notes” in Step 5 (Crosslinking of Chromatin).
• Consistently follow the manufacturer’s operational instructions and user-established chromatin shearing parameters to achieve reproducible shearing of chromatin in cell lysates.
• Replace the worn-out horn of the sonicator for a reproducible chromatin shearing profile. For each ChIP experiment, check the size distribution of sheared chromatin by running the sheared and cleaned-up DNA on an agarose gel along with a suitable DNA size marker. See Step 18 and associated notes for details.

### Problem 3

Low- and highly variable- ChIP Signal.

Low- and highly variable- ChIP signal can arise due to inconsistent crosslinking, low stability of the antigen-antibody complex to high ionic strength buffers, and proteolysis of the protein of interest. Excessive crosslinking can lead to epitope masking, difficulty in chromatin shearing, and less antigen availability in the soluble chromatin fraction. High ionic strength buffers, generally used in the washing steps of the ChIP experiment, can destabilize the antigen-antibody complex resulting in loss of immunoprecipitated DNA and a noisy ChIP signal. Moreover, the antigen of interest could undergo proteolysis during the immunoprecipitation and washing steps in the ChIP experiment.

### Potential solution

• To prevent excessive crosslinking, users should empirically determine the optimal crosslinking time for each crosslinker, preferably at a constant room temperature.
• For reliable and reproducible crosslinking, fresh (or minimally exposed to the environment) crosslinker (EGS and Formaldehyde in this protocol) stocks should be used. See “Critical notes” in Step 5 (Crosslinking of Chromatin) for details.
• The stability of the antigen-antibody complex to wash buffers should be empirically determined by the user beforehand.
• Maintaining the ChIP samples at 4°C and using protease inhibitors during the immunoprecipitation and in the wash buffers can minimize the proteolysis of the proteins. Aliquots of ChIP samples can be collected after steps 17, 23, 27, 29, 31, 35, and 39, and the stability of the protein of interest can be ascertained by western blotting using specific antibodies against the protein of interest.

## Resource availability

### Lead contact

Further information and requests for resources and reagents should be directed to and will be fulfilled by the lead contact, Mo Motamedi (mmotamedi@hms.harvard.edu).

### Technical contact

Technical questions on executing this protocol should be directed to and will be answered by the technical contact, Jasbeer S. Khanduja (jsk.acad1@gmail.com).

### Materials availability

This study did not generate new unique reagents.

### Data and code availability

This study did not generate new datasets or code.

## Acknowledgments

This work was supported by a National Institutes of Health grant (GM125782), an American Cancer Society Research Scholar Grant (18-056-01-RMC), a V Scholar grant, and a Ludwig Center at Harvard grant to M.M.

## Author contributions

J.S.K.: protocol development, optimization, experimentation and data analysis, manuscript conceptualization, writing, editing, and final approval. M.M.: supervision, funding, manuscript conceptualization, editing, and final approval.

## Declaration of interests

The authors declare no competing interests.
